# Supplementary material for: Learning curve and surgical outcome of robotic assisted colorectal surgery with ERAS program
Source: Sci Rep. 2022 Nov 29;12:20566. doi: 10.1038/s41598-022-24665-w (PMC9709162; doi:10.1038/s41598-022-24665-w)
Supplement: Supplementary file 1 — Supplementary Information 1. [file 41598_2022_24665_MOESM1_ESM.zip › LSC Raw data-Ñ[▒K/126/O_ñw╝╨░O▒Kñσ.pdf]

正式摘要(電子簽章：000000216561465)

W55 / W55

臺中榮民總醫院

Operation Note

[redacted]

- 手術日期：2021/05/06
- 手術醫師：VS 蔣鋒帆，CR 江世偉，CR 楊曙榮，R 古朋育，R 王鈺智
- Anesthesia : GE

Diagnosis

- Pre-operative diagnosis : Malignant tumor of rectum PENDING OBSTRUCTION
- Post-operative diagnosis : Ditto.

Surgical Indication

- For complete tumor resection and solve bowel obstruction

Operation

- 1. Radical laparoscopic anterior resection with end-to-end anastomosis (conversion to open), ILS 29mm, bilateral doughnuts: intact, re-inforcment with silk: nil. Air leak test: done, no air leak.
  2. High ligation (-) below left colic artery.
  3. Cut margin: proximal 10cm, distal 6cm.
  4. Take down of splenic flexure: nil.
  5. Reperitonealization: nil.
  6. One 12mm penrose drain at pelvis.
  7. Segmental resection with side to side anastomosis with lineal stapler
  8. Appendectomy was done
  9. Partial cystectomy due to tumor involvement

Finding

- 1. One 8 x 8 cm Borrmann's type III ulcerative tumor at recto-sigmoid junction colon, which invaded to the muscular layer, and penetrate through the serosa, urinary bladder, appendix and 1 segement of small bowel were involved . cT4N2M0.
  2. There were some enlarged indurated lymph nodes noted at pararectal region.
  3. There was no palpable liver metastasis, no visible peritoneal seeding.

Procedure

- After GA, the patient was placed in the modified Trendelenburg lithotomy position. Preoperative antibiotics were given. A Foley catheter and nasogastric tube were placed. The abdomen and perineum was prepped and draped in standard sterile fashion. A small vertical incision, about 1 cm, was made near the umbilicus and then a 12mm trochar was inserted into peritoneal cavity by open method. Pneumoperitoneum was established with CO2 inflation. Laparoscopy was inserted and thr other 12mm trochars were inserted over RLQ of abdomen. Three 5mm trochars were inserted at RUQ and LLQ of abdomen. Conversion to open due to small bowel involvement was done. A lower midline incision was made from just above the umbilicus to just above the pubis. This was deepened through the subcutaneous tissues and hemostasis was achieved with electrocautery. The linea alba was identified and incised and the peritoneal cavity entered. The abdomen was explored. Wound retractor was set up. The operative findings were mentioned as above. The small bowel was inspected and retracted to the right using a moist pad and Self-Retaining retractor. We resected small bowel and appendix which involved with RS-colon tumor.Using electrocautery, the colon was freed from its peritoneal attachments along the line of Toldt proximally from the descending colon and distally to the pelvic inlet. The peritoneum overlying the mesentery was then scored with electrocautery and the inferior mesentery artery was identified, ligated below left colic artery with double 2-0 silks, and transected, so was inferior mesentery vein. The plane anterior to the aorta was

- entered and colon, mesenteric fat were mobilized from retroperitoneum overlying the sacral promontory and into the pelvis. The incision was extended laterally and medially across the peritoneal reflection. The mesorectum was then mobilized using sharp and blunt dissection. Care was taken not to enter the presacral venous plexus. The rectum was carefully elevated until an adequate distal margin was noted. A linear stapler was then used to divide the rectum. Proximal and distal ends of the bowel were tagged and the specimen removed and sent to pathology. The mesenteric fat was gently cleared from the terminal 2-3 mm of the bowel ends. The field was isolated with moist pad and the bowel opened and sized. It was determined that a 29 mm circular stapler could be accommodated. A pursestring suture of 2-0 Prolene was placed, the anvil was inserted, and then the pursestring suture was tied. The anal canal was irrigated with distil water and then the circular stapler was introduced through the anus and pierced through the midportion of the stapler line. The proximal bowel with anvil in place was engaged with the circular stapler and the stapler was then fired. Two complete donuts were obtained and submitted to pathology. The anastomosis was tested for leaks with irrigation solution and air introduced through the rectum and was found to be patent and intact. The abdomen cavity was then copiously irrigated and hemostasis was checked again and a opened suction was placed in the pelvis and brought out through separate stab wounds. We consulted GU doctor re checking urinary bladder, they performed repaired urinary bladder. The fascia was closed with a running suture of No.1 Vicryl. The skin was approximated with 3-0 Nylon. The patient tolerated the procedure well and was brought to POR in stable condition
- Estimated blood loss: minimum
- 器械數量: 正確
- 紗布數量: 正確

Image

- PAINT
  - abdomen

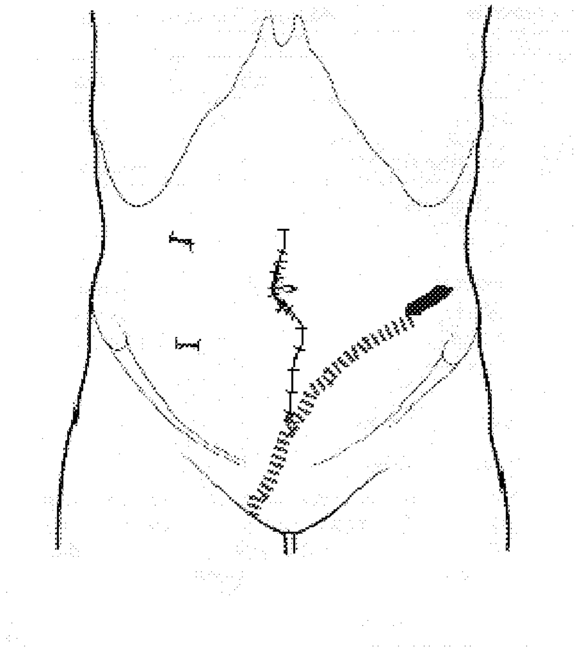

  - colon & rectum

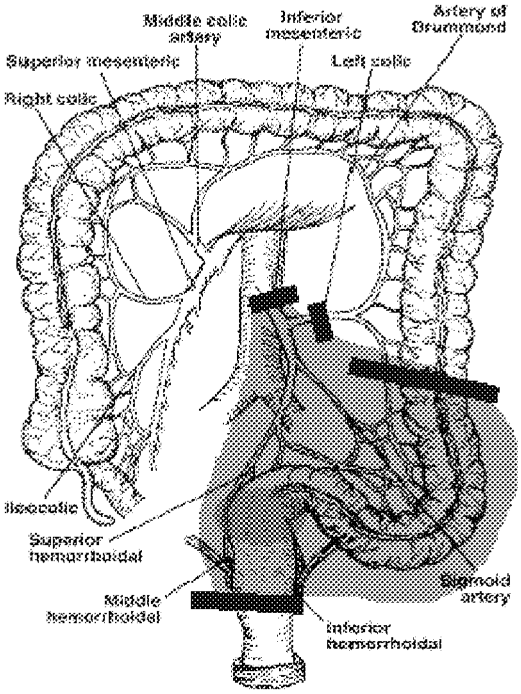

編輯主任 2062F蔣鋒帆、主治 2288K江世偉

正式摘要(電子簽章：**000000216561465)**

W55 / W55

doctorRank:B
